# Casticin induces ovarian cancer cell apoptosis by repressing FoxM1 through the activation of FOXO3a

**DOI:** 10.3892/ol.2013.1258

**Published:** 2013-03-14

**Authors:** LING JIANG, XIAO-CHENG CAO, JIAN-GUO CAO, FEI LIU, MEI-FANG QUAN, XI-FENG SHENG, KAI-QUN REN

**Affiliations:** 1 Department of Gynaecology and Obstetrics, The People’s Hospital of Hunan Province, First Affiliated Hospital of Hunan Normal University, Changsha 410005;; 2 Laboratory of Medicine Engineering, Medical College, Hunan Normal University, Changsha 410013, P.R. China

**Keywords:** ovarian cancer, casticin, FOXO3a, forkhead box protein M1, apoptosis

## Abstract

Casticin, a polymethoxyflavone, is reported to have anticancer activities. The aim of the present study was to examine the molecular mechanisms by which casticin induces apoptosis in ovarian cancer cells. The human ovarian cancer cell lines SKOV3 and A2780 were cultured *in vitro*. Various molecular techniques, including histone/DNA enzyme-linked immunosorbent assay (ELISA), reverse transcription polymerase chain reaction (RT-PCR), western blot analysis and gene transfection, were used to assess the expression of FOXO3a and forkhead box protein M1 (FoxM1) in casticin-treated ovarian cancer cell lines. Casticin-induced apoptotic cell death was accompanied by the activation of transcription factor FOXO3a, with a concomitant decrease in the expression levels of FoxM1 and its downstream target factors, namely survivin and polo-like kinase 1 (PLK1), and an increase in p27^KIP1^. A small inhibitory RNA (siRNA) knockout of FoxM1 potentiated casticin-induced apoptosis in ovarian cancer cells. Silencing FOXO3a expression using siRNA increased FoxM1 expression levels and clearly attenuated the induction of apoptosis by casticin treatment. These results show that casticin-induced apoptosis in ovarian cancer may be caused by the activation of FOXO3a, leading to FoxM1 inhibition.

## Introduction

Ovarian cancer is the most common cause of cancer-associated mortalities arising from gynecological tumors (1,2). The most common treatment approach for ovarian cancer consists of a combination of surgery and chemotherapy. Over the past three decades, surgical tumor debulking followed by platinum-based chemotherapy has been the standard treatment for advanced ovarian cancer. Although response rates and complete responses in advanced disease after first-line treatment with carboplatin and paclitaxel are >80% and 40–60%, respectively, the majority of patients eventually relapse, with a median progression-free survival of 18 months (3). Therefore, there is an urgent demand to test novel drugs for the prevention and treatment of ovarian cancer.

Casticin is one of the main components of the fruit of *Vitex rotundifolia L.* Casticin has been shown to exert an anti-inflammatory effect *in vivo*(4) and has been widely used in traditional Chinese medicine as an anti-inflammatory drug for thousands of years. Increasing numbers of studies have shown that casticin exhibits anticarcinogenic activity in breast (5), cervical (6,7), lung and colon cancer (8–10) and hepatocellular carcinoma (11), in addition to ovarian cancer (12). It has been proposed that cell cycle arrest and casticin-induced apoptosis may be the possible mechanisms of its anticancer effects. However, the precise underlying mechanisms are not fully elucidated.

The forkhead box protein M1 (FoxM1) belongs to a family of evolutionarily conserved transcriptional regulators that are characterized by the presence of a DNA-binding domain called the forkhead box or winged-helix domain (13,14). Numerous studies have demonstrated the biological significance of FoxM1 in controlling tumor aggressiveness. FoxM1 has been shown to be involved in cell proliferation and apoptosis, which affects the developmental function of several organs (14,15). A number of studies have revealed FoxM1 to be a key cell cycle regulator in the transition from G1 to S phase and in the progression to mitosis (16,17). The loss of FoxM1 expression leads to mitotic spindle defects, the mitotic delay of cells and induction of mitotic catastrophe or apoptotic cell death (18–20). FoxM1 has also been shown to regulate the transcription of cell cycle genes essential for G1-S and G2-M progression, including survivin, Cdc25B, cyclin B, cyclin D1, p21^CIP1^ and p27^KIP1^(21–23). FoxM1 has been shown to bind to the mammalian mitotic kinase polo-like kinase 1 (PLK1), thus acting as a mediator of the PLK1-dependent regulation of cell cycle progression (24,25).

FOXO3a is a member of the FOXO subfamily of fork-head transcription factors. The phosphorylation of FOXO3a results in the impairment of its DNA-binding ability and an increased binding affinity for the 14-3-3 proteins. This causes FoxM1 upregulation, which in turn promotes cell proliferation and survival (26–28). By contrast, the dephosphorylation of activated FOXO3a induces cell cycle arrest and apoptosis (29–31). Since casticin promotes cell cycle arrest and apoptosis, it is possible that it exerts these antitumor effects by regulating FOXO3a/FoxM1 expression. However, the intracellular mechanisms by which casticin induces apoptosis in ovarian cancer cells via the regulation of FOXO3a/FoxM1 signaling have never been examined. Hence, the aim of the present study was to examine the molecular mechanisms by which casticin-induced activation of the transcription factor FOXO3a induces apoptosis in ovarian cancer cells. Our results demonstrated that casticin promotes the dephosphorylation of FOXO3a, leading to the inhibition of FoxM1, which ultimately causes ovarian cancer cell apoptosis.

## Materials and methods

### Chemicals and antibodies

Casticin was purchased from Chengdu Biopurify Phytochemicals Ltd. (Chengdu, Sichuan, China). It has a molecular weight of 374.3, appears as yellow crystals and has a purity of 98.0%. Casticin was prepared in dimethyl sulfoxide (DMSO) as a 10 mmol/l stock solution and diluted in Dulbecco’s minimum essential medium (DMEM; Invitrogen, Carlsbad, CA, USA) to the indicated concentration before use. Primary antibodies FoxM1, PLK1, p27^KIP1^, survivin, β-actin, anti-caspase-3 and anti-poly (ADP-ribose) polymerase (PARP) were purchased from Santa Cruz Biotechnology, Inc. (Santa Cruz, CA, USA). Horseradish peroxidase-conjugated rabbit anti-mouse secondary antibody was also purchased from Santa Cruz Biotechnology, Inc. Mouse monoclonal antibodies against FOXO3a and phospho-FOXO3a-Thr^32^ were purchased from Millipore (Bedford, MA, USA). Lipofectamine™ 2000 was purchased from Invitrogen. Protease inhibitor cocktail and all other chemicals were obtained from Sigma (St. Louis, MO, USA).

### Cells and cell culture

The human ovarian cancer cell lines SKOV3 and A2780 were purchased from the China Center for Type Culture Collection (CCTCC; Wuhan, China). The cells were maintained in DMEM supplemented with 10% fetal bovine serum (FBS), 4 mM glutamine, 100 U/ml penicillin and 100 *μ*g/ml streptomycin and incubated at 37°C in a humidified atmosphere of 5% CO_2_.

### Histone/DNA enzyme-linked immunosorbent assay (ELISA) for detecting apoptosis

The cell apoptosis ELISA detection kit (Roche Applied Sciences, Penzberg, Germany) was used to detect apoptosis in casticin-treated cells, according to the manufacturer’s instructions. Briefly, cells were seeded in 96-well plates at a density of 1×10^4^ cells/well for 24 h and the test agents were added to the culture medium containing 10% FBS. After 24 h, the cytoplasm of cells in the control (untreated) and treatment (casticin-treated) groups was transferred to streptavidin precoated 96-well plates, which had been previously incubated with a biotinylated histone antibody and peroxidase-tagged mouse anti-human DNA for 2 h at room temperature. The absorbance was measured at 405 nm using an ELx800 ELISA plate reader (Bio-Tek, Winchester, VA, USA).

### Reverse transcription polymerase chain reaction (RT-PCR)

Total RNA was extracted using TRIzol^®^ reagent (Life Technologies, Gaithersburg, MD, USA). The integrity of the RNA was assessed using 2% agarose gel electrophoresis. RNA (∼2 *μ*g) was reverse transcribed using the SuperScript™ First-Strand Synthesis System kit (Invitrogen). cDNAs encoding the FoxM1 and glyceraldehyde-3-phosphate dehydrogenase (GAPDH) genes were amplified by PCR as follows: denaturation at 94°C for 30 sec, annealing at 63°C for 30 sec and elongation at 72°C for 45 sec. The primer sequences were designed as follows: for FoxM1, the forward primer was 5′-AAC CGCTACTTGACATTGG-3′ and the reverse primer was 5′-GCAGTGGCTTCATCTTCC-3′. A GAPDH housekeeping gene was used as the internal control, for which the forward primer was 5′-ACCCAGAAGACTGTGGATGG-3′ and the reverse primer was 5′-TGCTGTAGCCAAATTCGTTG-3′. PCR products were analyzed by 2% agarose gel electrophoresis.

### RNA interference

FoxM1, FOXO3a and control small interfering RNA (siRNA) were purchased from Santa Cruz Biotechnology, Inc. Human ovarian cancer SKOV3 and A2780 cells were transfected with FoxM1, FOXO3a and control siRNA using Lipofectamine™ 2000, as described by Wang *et al*(32). The cells were then collected and processed for western blot analysis and histone/DNA ELISA.

### Western blot analysis

Total cell extracts for western blot analysis were obtained, as previously described (33). Cell lysates containing 50 *μ*g protein were electrophoresed on a 7.5–12.5% sodium dodecyl sulfate polyacrylamide gel and blotted onto polyvinylidene difluoride membranes (Millipore). Anti-FoxM1, anti-p27^KIP1^, anti-survivin, anti-FOXO3a, anti-phospho-FOXO3a-Thr^32^, anti-caspase-3 and anti-PARP were used as primary antibodies. The blots were stripped and reprobed with an anti-actin antibody to normalize for differences in protein loading. Changes in the levels of the desired proteins were determined by densitometric scanning of the immunoreactive bands and corrected for β-actin loading control. Immunoblotting for each protein was performed at least twice using independently prepared lysates to ensure reproducibility of the results.

### Statistical analysis

The database was set up with the SPSS 15.0 software package (SPSS Inc., Chicago, IL, USA) for statistical analysis. Data are presented as mean ± standard deviation (SD). The means of multiple groups were compared using one-way analysis of variance (ANOVA) after the equal check of variance and comparisons between the means were performed using the least significant difference (LSD) method. Statistical comparison was also performed using a two-tailed t-test when appropriate. P<0.05 was considered to indicate a statistically significant difference.

## Results

### Effects of casticin on apoptosis in ovarian cancer cells

**Figure 1:**
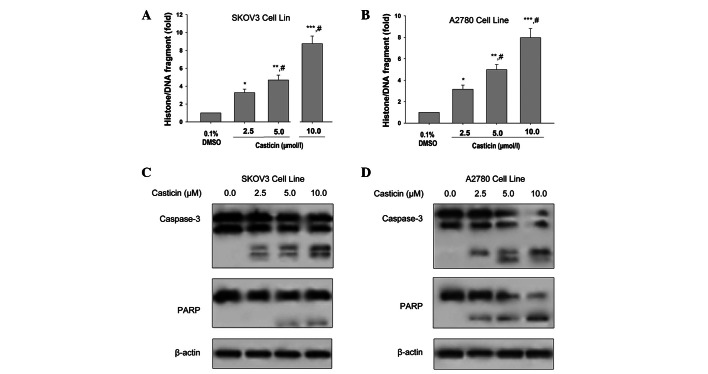
Effects of casticin on apoptosis in ovarian cancer cells. (A and B) SKOV3 and A2780 cells were treated with the indicated concentrations of casticin for 24 h. Histone/DNA fragmentation was determined using ELISA. Data are presented as mean ± standard deviation (SD; n=3). ^*^P<0.05, ^**^P<0.01 and ***P<0.001 vs. 0.1% dimethyl sulfoxide (DMSO); ^#^P<0.05 vs. treatment with 2.5 *μ*mol/l casticin. (C and D) Cell treatment was the same as in A and B. The expression levels of caspase-3 and poly (ADP-ribose) polymerase (PARP) were determined by western blot analysis in the total cell lysates and β-actin was used as the loading control. ELISA, enzyme-linked immunosorbent assay; SD, standard deviation.

Based on results from studies that demonstrated the casticin-induced growth inhibition of ovarian cancer cells (12) and apoptosis in human cervical cancer (6,7) and hepatocellular carcinoma (11) cells, we first investigated whether casticin was able to induce apoptosis in ovarian cancer cells. Exposing SKOV3 and A2780 cells to 2.5, 5.0 and 10.0 *μ*mol/l casticin for 24 h significantly induced histone/DNA fragmentation in a concentration-dependent manner (Fig. 1A and B). Additionally, casticin (2.5, 5.0 and 10.0 *μ*mol/l) activated caspase-3 and induced the cleavage of its substrate, PARP, in SKOV3 and A2780 cells. Substrate cleavage was indicated by a reduction in the uncleaved forms of caspases and their substrates and/or the appearance of their cleaved forms (Fig. 1C and D). Taken together, these results demonstrated that casticin induces apoptosis in ovarian cancer cells.

### Effects of casticin on FoxM1 expression in ovarian cancer cells

**Figure 2:**
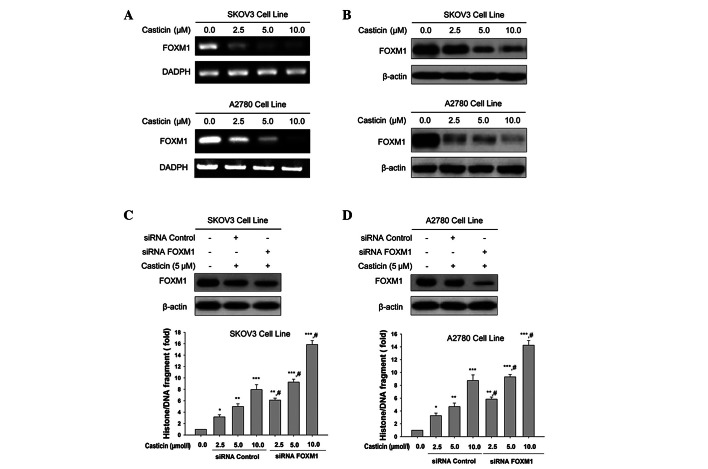
Effects of casticin on the expression of FoxM1 in ovarian cancer cells. (A and B) SKOV3 and A2780 cells were treated with the indicated concentrations of casticin for 24 h. The expression of FoxM1 was determined using (A) RT-PCR and (B) western blot analysis. (C and D) SKOV3 and A2780 cells were transiently transfected with a control non-specific siRNA or FoxM1-specific siRNA for 48 h, followed by treatment with the indicated concentrations of casticin for 24 h. The expression levels of caspase-3 and PARP were determined by western blot analysis in the total cell lysates and β-actin was used as the loading control. Histone/DNA fragmentation was determined using ELISA. Data are presented as mean ± SD (n=3). ^*^P<0.05, ^**^P<0.01 and ***P<0.001 vs. 0.1% DMSO; ^#^P<0.05 vs. cells treated with the same concentration of casticin before being transfected with non-specific siRNA. FoxM1, forkhead box protein M1; RT-PCR, reverse transcription polymerase chain reaction; siRNA, small inhibitory RNA; PARP, poly (ADP-ribose) polymerase; ELISA, enzyme-linked immunosorbent assay; SD, standard deviation; DMSO, dimethyl sulfoxide.

Studies have shown that the loss of FoxM1 expression induces apoptosis (19). We investigated whether casticin was able to regulate FoxM1 expression during casticin-induced apoptosis in ovarian cancer cells. The expression of FoxM1 was determined using RT-PCR and western blot analysis. We showed that FoxM1 was overexpressed in SKOV3 (Fig. 2A) and A2780 (Fig. 2B) cell lines. Exposing SKOV3 and A2780 cells to 2.5, 5.0 and 10.0 *μ*mol/l casticin for 24 h significantly reduced the expression of FoxM1 at the protein level (Fig. 2A and B). Silencing FoxM1 expression using siRNA resulted in the enhanced induction of apoptosis with casticin treatment in SKOV3 (Fig. 2C) and A2780 (Fig. 2D) cell lines.

### Effects of casticin on the expression of downstream targets of FoxM1 in ovarian cancer cells

**Figure 3:**
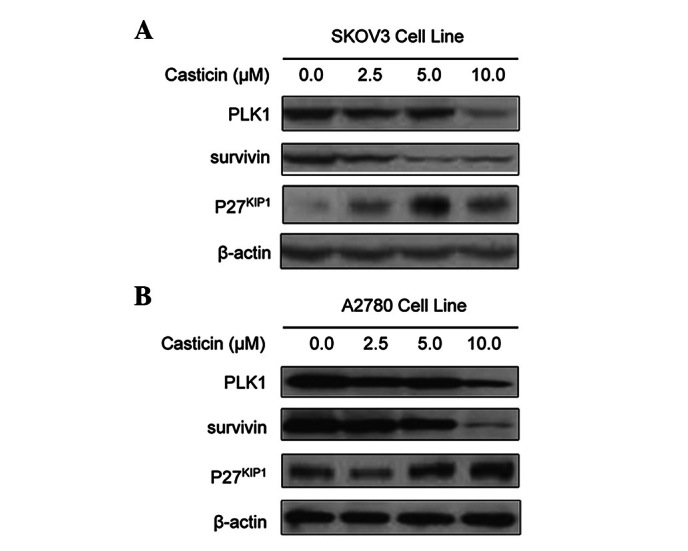
Effects of casticin on the expression of FoxM1 downstream targets in ovarian cancer cells. (A) SKOV3 and (B) A2780 cells were treated with the indicated concentrations of casticin for 24 h. The expression of PLK1, survivin and p27^KIP1^ was analyzed using western blot analysis. β-actin was used as a loading control. FoxM1, forkhead box protein M1; PLK1, polo-like kinase 1.

To further confirm the effects of casticin on the functional regulation of FoxM1, we assessed the expression of FoxM1 downstream target genes in SKOV3 and A2780 cells following casticin treatment. FoxM1 is known to have several downstream target genes, including PLK1, survivin and p27^KIP1^(21,34–36). Western blot analysis revealed that casticin reduced the expression levels of PLK1 and survivin and increased expression of p27^KIP1^ at the protein level in SKOV3 (Fig. 3A) and A2780 (Fig. 3B) cells. These results provide molecular evidence suggesting that casticin-induced apoptosis in ovarian cancer cells may be mediated via the inactivation of FoxM1.

### Effects of casticin on the phosphorylation level of FOXO3a protein in ovarian cancer cells

**Figure 4:**
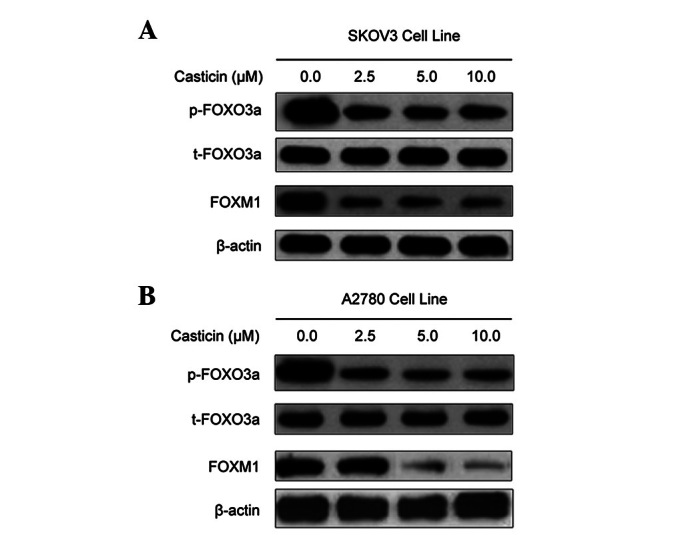
Effects of casticin on the phosphorylation level of FOXO3a protein in ovarian cancer cells. (A) SKOV3 and (B) A2780 cells were treated with the indicated concentrations of casticin for 24 h. The expression of phosphorylated FOXO3a (p-FOXO3a), total FOXO3a (t-FOXO3a) and FoxM1 was analyzed using western blot analysis. β-actin was used as a loading control. FoxM1, forkhead box protein M1.

Since FOXO3a is considered to be an upstream regulator of the FoxM1 transcription factor (29), we investigated the expression of phosphorylated FOXO3a protein in order to explain the mechanism of casticin-dependent FoxM1 inhibition. Western blot analysis showed that treatment with casticin led to a decrease in the FOXO3a phosphorylation level, with a corresponding decrease in FoxM1 expression levels (Fig. 4). These results indicate that casticin-mediated inhibition of FoxM1 expression may be associated with the inhibition of FOXO3a phosphorylation.

### Effects of silencing the FOXO3a gene on casticin-mediated apoptosis in ovarian cancer cells

**Figure 5:**
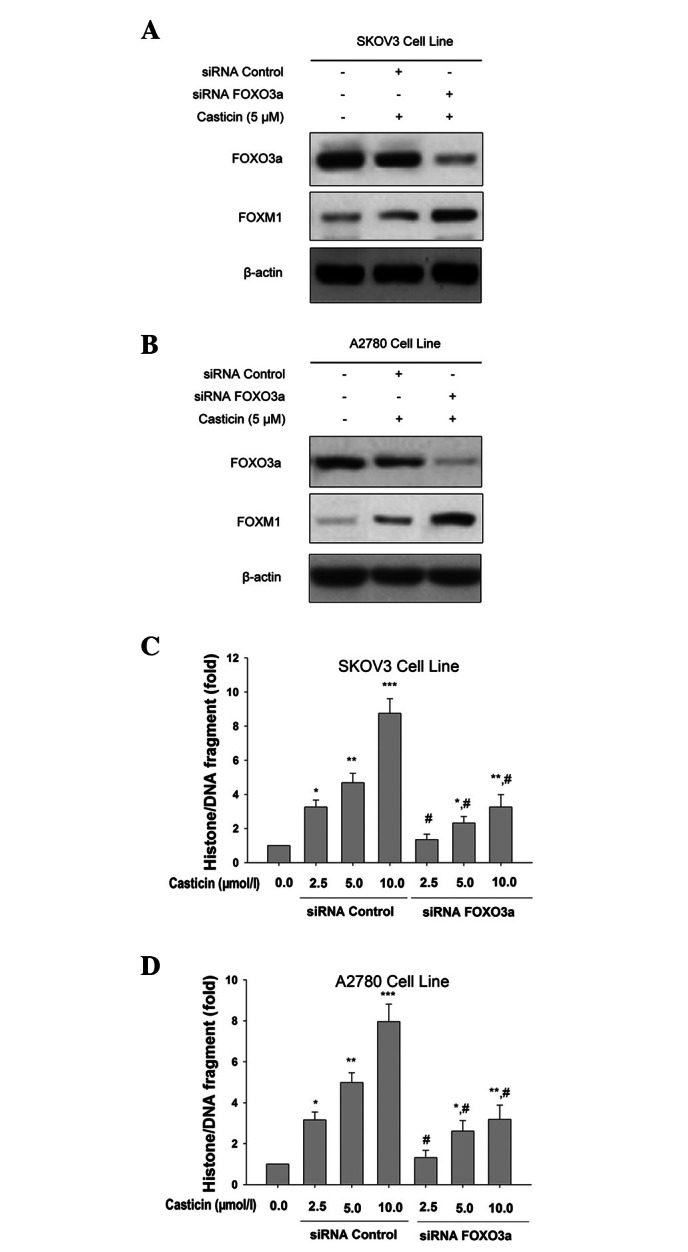
Effects of silencing the FOXO3a gene on casticin-mediated apoptosis in ovarian cancer cells. (A and B) SKOV3 and A2780 cells were transiently transfected with a control non-specific siRNA or FOXO3a-specific siRNA for 48 h, followed by treatment with DMSO or casticin at the indicated concentrations for 24 h. The expression of FOXO3a and FoxM1 was analyzed using western blot analysis. β-actin was used as a loading control. (C and D) SKOV3 and A2780 cells were transiently transfected with a control non-specific siRNA or FOXO3a-specific siRNA for 48 h, followed by treatment with DMSO or casticin at the indicated concentrations for 24 h. Histone/DNA fragmentation was determined using ELISA. Data are presented as mean ± SD (n=3). ^*^P<0.05, ^**^P<0.01, ^***^P<0.001 vs. 0.1% DMSO; ^#^P<0.05 vs. cells treated with the same concentration of casticin before being transfected with non-specific siRNA. siRNA, small inhibitory RNA; DMSO, dimethyl sulfoxide; ELISA, enzyme-linked immunosorbent assay; FoxM1, forkhead box protein M1; SD, standard deviation.

In order to confirm the role of the FOXO3a transcription factor in the cellular response to casticin, we performed gene silencing experiments. SKOV3 and A2780 cells were generated and expression of the FOXO3a protein was attenuated using siRNA technology. In FOXO3a siRNA-transfected cells, the expression levels of FoxM1 clearly increased (Fig. 5A and B). Additionally, we identified that the knockdown of FOXO3a significantly attenuated casticin-induced apoptosis in ovarian cancer cells (Fig. 5C and D). These findings are consistent with our hypothesis that casticin induces ovarian cancer cell apoptosis by repressing FoxM1 expression through the induction of FOXO3a activity.

## Discussion

In the present study, we showed that casticin induced apoptosis in ovarian cancer cells occurs due to a decrease in the expression levels of FoxM1 and its downstream targets PLK1 and survivin and an increase in p27^KIP1^, all of which are associated with activation of the FOXO3a transcription factor via dephosphorylation. Additionally, silencing the FOXO3a transcription factor using an siRNA approach blocked casticin-induced downregulation of FoxM1 and inhibited apoptosis. Our results suggest that casticin induces apoptosis in ovarian cancer cells (SKOV3 and A2780) through the regulation of FOXO3a/FoxM1 signaling.

A number of studies have demonstrated overexpression of the FoxM1 gene in human cancer cells and tissues, including in ovarian cancer (32,33,37,38), and emerging evidence suggests that the inactivation of FoxM1 may have important implications in cancer therapy. For example, it may be possible to downregulate FoxM1 expression using specific drugs, such as siomycin A, thiostrepton and the epidermal growth factor receptor (EGFR) inhibitor gefitinib (27,39,40). Wang *et al* demonstrated that genistein is capable of inhibiting FoxM1 activation in pancreatic cancer cells, leading to cell growth inhibition and the induction of apoptosis (32). In the present study, we investigated the possibility that casticin induces apoptotic cell death and aimed to determine the role of FoxM1 in casticin-dependent ovarian cancer cell apoptosis. Our data showed that casticin elicited a marked effect on apoptosis in ovarian cancer cells, as demonstrated by histone/DNA ELISA results which showed the activation of caspase-3 and cleavage of PARP, accompanied by the downregulation of FoxM1 expression. Our results suggest that FoxM1 is a target of casticin in ovarian cancer cells, as FoxM1 is known to induce oncogenesis and its downregulation causes the inhibition of cell growth (40).

The FOXO3a transcription factor is important in the regulation of the cell cycle and apoptosis (41,42). FOXO3a is regulated by phosphorylation (43). Upon activation of the PI3K/AKT signaling pathway, FOXO proteins undergo AKT-mediated phosphorylation, which promotes their binding to 14-3-3 proteins and facilitates their nuclear export through chromosome region maintenance 1 (CRM1) and cytoplasmic sequestration. Under conditions of stress or in the absence of growth or survival factors, the PI3K/AKT pathway is inhibited and FOXO3a proteins translocate to the cell nucleus, where they execute their transcriptional functions (40). In the present study, we demonstrated that casticin inhibited FOXO3a phosphorylation. Furthermore, knockdown of FOXO3a by transfection with siRNA blocked the casticin-induced down-regulation of FoxM1 expression and inhibited ovarian cancer cell apoptosis. These findings indicate that FOXO3a is a key regulator of casticin-induced apoptosis and FoxM1 expression in ovarian cancer cells.

In summary, our study demonstrated that casticin-induced dephosphorylation of FOXO3a regulates the expression of FoxM1 and its target genes, including survivin, PLK1 and p27^KIP1^, and this causes apoptosis in ovarian cancer cells. Further studies are required to assess the upstream events leading to FOXO3a phosphorylation and casticin-dependent anticancer effects in ovarian cancer cells. A thorough understanding of the mechanisms and effects of casticin on the cell cycle and apoptosis may lead to the identification and development of novel therapeutic molecules for the treatment and prevention of ovarian cancer and other malignant diseases.
